# The Role of PAX7 in Breast Cancer Prognosis and Its Mechanistic Involvement in the Wnt/β‐Catenin Pathway

**DOI:** 10.1111/jcmm.70602

**Published:** 2025-05-15

**Authors:** Qidong Ge, Wei Zhang, Chao Li, Xinlin Li, Zhen Wang, Xujun Li

**Affiliations:** ^1^ Department of Oncology Ningbo No. 2 Hospital Ningbo Zhejiang People's Republic of China; ^2^ Department of Breast Surgery Ningbo No. 2 Hospital Ningbo Zhejiang People's Republic of China; ^3^ Department of Anesthesiology The First Affiliated Hospital of Ningbo University Ningbo Zhejiang People's Republic of China

**Keywords:** metastasis, PAX7, prognosis, proliferation, Wnt/β‐catenin pathway

## Abstract

Breast cancer significantly affects women's lives globally. While PAX7 (Paired Box 7), a regulatory protein linked to muscle growth, has been connected to various cancers, its role in breast cancer is not well understood. This study explores PAX7's significance in breast cancer and its mechanisms. RNA‐seq data from the TCGA database assessed PAX7 expression across cancer types. Prognostic value in breast cancer was evaluated using Kaplan–Meier and Cox regression analyses. Functional experiments, including high‐throughput sequencing, cell growth analysis, colony formation, Transwell assays, and Western blot analysis, were conducted on PAX7 knockdown cell lines (MDA‐MB‐468 and MDA‐MB‐231). Results showed high PAX7 expression in breast cancer linked to lower survival rates. PAX7 knockdown affected over 2000 genes and inhibited cancer cell proliferation, migration, and invasion, involving the Wnt/β‐catenin pathway. SKL2001 reversed these effects. PAX7 is a potential prognostic biomarker and therapeutic target, with elevated levels indicating a poor prognosis. Further research on PAX7‐targeted therapies is needed.

## 1 Introduction

Breast cancer is a prevalent form of cancer in women globally, impacting 1 out of every 20 women worldwide and 1 out of every 8 women in affluent nations [1, 2]. Despite the advancements in mammography and adjuvant therapy leading to better survival rates for breast cancer patients in recent years, the number of cases continues to rise worldwide [3]. In terms of breast cancer research progress, in recent years, the focus has been on in‐depth research on molecular mechanisms and the development of new treatment strategies [2, 4]. Targeted therapy and immunotherapy are current research hotspots. The outlook for patients with HER2‐positive breast cancer has greatly improved through the use of targeted therapy like trastuzumab [1, 4, 5, 6]. Regarding immunotherapy, the use of PD‐1/PD‐L1 inhibitors in triple‐negative breast cancer has demonstrated some effectiveness, yet additional studies are required to determine the best approach and suitable patient population [1, 2, 4, 5, 6]. Thus, understanding the intricate and varied epidemiological and pathological features of breast cancer underscores the importance of discovering novel biomarkers to enhance the diagnosis, treatment, and prognosis of this disease.

PAX7, a member of the PAX gene family, functions as a transcription factor [7, 8, 9]. This particular gene is crucial in the process of embryonic development, particularly in the formation of the nervous and muscular systems [7, 8, 10, 11]. Anomalous PAX7 levels have been linked to several types of cancer, particularly rhabdomyosarcoma, where the fusion of the PAX7 gene with the FOX01 gene (known as the PAX7‐FOXO1 fusion gene) is a frequent genetic occurrence [8, 12]. PTEN in glioma binds to the PAX7 promoter, suppressing PAX7 transcription through interaction with cAMP response element binding protein 1 (CREB)/CREB binding protein (CBP) [13]. Nevertheless, there has been limited research on the involvement of PAX7 in breast cancer.

The Wnt/β‐catenin signalling pathway is a highly conserved mechanism that plays a key role in embryonic development and tissue homeostasis, and is closely related to the occurrence and development of various cancers [14, 15, 16]. In the classical Wnt/β‐catenin signalling pathway, Wnt ligands bind to Frizzled receptors and LRP5/6 coreceptors, activate downstream signalling cascades, inhibit the activity of degradation complexes (Axin, APC, GSK‐3β), allow β‐catenin to accumulate and translocate into the nucleus, bind to TCF/LEF to regulate gene expression, and thus affect cell proliferation, differentiation, and migration [14, 15, 16, 17, 18]. In breast cancer, the Wnt/β‐catenin signalling pathway is closely related to tumour cell proliferation, invasion, drug resistance, and maintenance of cancer stem cells (BCSCs) [14, 16, 17, 19]. Studies have shown that inhibiting Wnt signals can reduce the metastasis and drug resistance of breast cancer cells. For example, ZFP57 inhibits Wnt/β‐catenin signals and reduces cancer cell proliferation and invasion by regulating the methylation of the MEST gene [20]. At the same time, this pathway is also closely related to the tolerance of chemotherapy and radiotherapy, and the development of drugs targeting Wnt signals has become a potential therapeutic strategy. Therefore, molecular regulation of the Wnt/β‐catenin signalling pathway is an important direction for breast cancer treatment.

Our research group revealed the differential expression of PAX7 in seven tumours through analysis of TCGA database RNA‐seq data. PAX7 shows high expression levels in breast cancer (BRCA), lung adenocarcinoma (LUAD), lung squamous cell carcinoma (LUSC), gastric adenocarcinoma (STAD), and thyroid cancer (THCA), while also being highly expressed in prostate adenocarcinoma (PRAD) and glioma (GBM). The Kaplan–Meier analysis indicated a correlation between PAX7 levels and the prognosis of breast cancer. Elevated levels of PAX7 in breast cancer patients were linked to lower overall survival rates, while no notable prognostic correlation was found in glioma, lung adenocarcinoma, lung squamous cell carcinoma, or gastric adenocarcinoma. The nomogram, derived from Cox regression analysis, indicated that PAX7 is highly precise in forecasting the 1‐year, 3‐year, and 5‐year overall survival rates of patients with BRCA. By constructing two PAX7 knockdown stably transduced cell lines and performing high‐throughput sequencing analysis, we found that the knockdown of PAX7 caused significant changes in gene expression, affecting more than 2000 genes. Functional enrichment analysis revealed that the differential genes are primarily associated with the abilities of cell proliferation and adhesion. Experiments involving Western blot, colony formation, and Transwell assays demonstrated that the suppression of PAX7 hindered the growth, infiltration, and movement of breast cancer cells by blocking the Wnt/β‐catenin pathway. Therefore, PAX7 may be a promising biomarker and potential therapeutic target in breast cancer, where high expression is associated with poor prognosis. Inhibition of PAX7 disrupts cancer cell proliferation and metastasis.

## 2 Materials and Methods

### 2.1 Data Sources

Data on 33 types of cancer from the TCGA (The Cancer Genome Atlas) (https://portal.gdc.cancer.gov/) and GTEx (Genotype‐Tissue Expression) (https://gtexportal.org/) databases were compiled [21, 22]. The research adhered to the data usage rules and ethical standards set by TCGA and GTEx, with thorough data processing and analysis conducted to guarantee the precision and dependability of the study findings.

### 2.2 Prognostic Analysis and Prognostic Model Generation

In our study, we assessed prognostic factors using Cox regression analysis and the Kaplan–Meier method. Multivariable Cox analysis was conducted to assess the impact of PAX7 expression on survival concerning other clinical characteristics. The threshold for PAX7 expression was established using the median, with statistical significance indicated by a *p‐*value less than 0.05. ROC analysis was conducted with the pROC package to assess the diagnostic effectiveness of PAX7 expression in distinguishing BRAC patients from healthy samples [23]. AUC scores vary between 0.5 and 1.0, representing a detection accuracy of 50%–100%. Personalised prediction of overall survival (OS) in BRAC patients using the RMS R package. To assess model calibration, we generated a calibration curve by plotting the ratio of predicted probabilities to observed rates in a nomogram. We conducted an extensive analysis comparing the predictive precision of nomograms versus single prognostic factors through the assessment of the C‐index and the creation of receiver operating characteristic (ROC) curves. Statistically significant results were defined as *p* < 0.05.

### 2.3 Cell Culture and Establishing Stable Transfection Cell Lines

The interference sequence of shPAX7‐1^#^ is 5′‐GCTCAGAATCAAGTTCGGGAA‐3′, and the interference sequence of shPAX7‐2^#^ is 5′‐CTGCTTGTTTATGGAG AGCTA‐3′. The lentiviral vector plasmid was combined with the packaging plasmid PMD2.G and psPAX7, and transfected into 293T cells using Lipofectamine 3000 transfection reagent (L3000150, Thermo Fisher). The viral supernatant was collected after 48 and 72 h, respectively, and filtered with a 0.22 μm filter. The MDA‐MB‐231 and MDA‐MB‐468 cells were infected with the viral supernatant for 24 h, the culture medium was replaced, and puromycin was selected until stable survival.

### 2.4 Total RNA Extraction

MDA‐MB‐231 were placed in 6‐well plates with 3 × 10^5^ cells per well and subjected to the specified experimental conditions. Samples were centrifuged to collect cells, which were then resuspended in 1 mL of Trizol reagent (Beyotime, Shanghai, China) and subsequently stored in liquid nitrogen. Samples were sent to Oebiotech company (China)for RNA sequencing (RNA‐seq).

### 2.5 RNA‐Seq Analysis

After RNA quality detection, RNA fragmentation, reverse transcription to generate cDNA, PCR amplification, and other steps, the raw data is obtained by sequencing, and then through sequencing quality assessment and preprocessing, the protein‐coding gene expression is obtained by genome alignment. DESeq2 package was utilised to analyse differential expression between the two distinct groups. Genes with a |log2(FC)| ≥ 1 and *p‐*adj < 0.05 were identified as differentially expressed. Functional and pathway differences between different groups were illustrated by performing gene set enrichment analysis (GSEA), gene ontology (GO), and Kyoto Encyclopedia of Genes and Genomes (KEGG) pathway enrichment analysis with the R package ClusterProfiler [24].

### 2.6 Cell Proliferation Experiment

The cells to be tested were seeded in a 96‐well plate. Cultivated at 37°C with 5% CO_2_ for durations of 0,24, 48, 72, and 96 h. Following the guidelines provided by the CCK‐8 kit (Bioss item number BA00208), 10 μL of CCK‐8 solution was introduced into every well. Gently shake the 96‐well plate to evenly distribute the CCK‐8 solution. Utilise a microplate reader to determine the absorbance (OD value) of every well at a 450 nm wavelength.

Dilute the cell suspension and inoculate it into a 6‐well plate, with 1000 cells in each well. Incubate the inoculated cells at 37°C with 5% CO_2_ for a period of 10 days. Add an appropriate amount of 4% paraformaldehyde solution to each well and fix the cells at room temperature for 10–15 min. After decanting the fixative, rinse the cells with PBS two times. Add 0.1% crystal violet staining solution and stain at room temperature for 15–30 min. Pour off the staining solution and rinse gently with running water until the background is clear and there is no excess dye. Once the cells have been stained, they should be examined using an inverted microscope to determine the number of cell clones present in each well.

### 2.7 Western Blot

Total cell protein was obtained by lysing cell samples on ice using RIPA lysis buffer with PMSF from Servicebio in China. The BCA protein assay kit from Servicebio, China, was utilised to measure the protein levels in the samples. The target proteins were separated by SDS‐PAGE gel electrophoresis. Following electrophoresis, the proteins were moved from the gel onto a PVDF membrane. The membranes were obstructed using 5% skim milk powder for one hour at room temperature, then exposed to primary antibodies overnight at 4°C. The next day, the corresponding secondary antibodies were incubated for 1 h at room temperature. Finally, the target protein bands were visualised using an enhanced chemiluminescence kit (Servicebio, China). The detailed information on the primary and secondary antibodies in the experiment is as follows: GAPDH (Proteintech, 60004‐1‐Ig), PAX7 (Proteintech, 20570‐1‐AP), c‐MYC (Proteintech, 10828‐1‐AP), Bete Catenin (Proteintech, 51067‐2‐AP), Bete Catenin (Proteintech, 51067‐2‐AP), Cyclin D1 (Proteintech, 60186‐1‐Ig).

### 2.8 Real‐Time PCR

Quantitative real‐time RT‐PCR (qRT‐PCR) analysis was used to measure mRNA levels with the assistance of PerfectStart Green qPCR SuperMix (TransGen, China) and LightCycler 96 System (Roche, Switzerland). The primers used for real‐time PCR are as follows:

- GAPDH forward Primer: 5′‐ACAACTTTGGTATCGTGGAAGG‐3′.
- reverse Primer: 5′‐GCCATCACGCCACAGTTTC‐3′.
- PAX7 forward Primer: 5′‐ACCCCTGCCTAACCACATC‐3′.
- reverse Primer: 5′‐GCGGCAAAGAATCTTGGAGAC‐3′.

### 2.9 Transwell Assay

The Transwell test assesses cell migration or invasion by placing a cell suspension in the top chamber and 20% FBS culture medium in the bottom chamber. Following a 48‐h incubation at 37°C with 5% CO_2_, the cells that did not migrate were removed, while those that migrated or invaded the lower chamber were fixed using 4% paraformaldehyde and stained with 0.1% crystal violet. Afterward, the cells with stains were observed under a microscope to assess their ability to migrate or invade.

### 2.10 Statistical Analysis

Statistical analysis and graphics were performed using R software (version 4.4.2). One‐way analysis of variance (ANOVA) was used for intergroup comparisons. Statistical significance was defined as *p* < 0.05. We used Cox regression analysis and the Kaplan–Meier method to evaluate prognostic factors in our study. To compare the effect of PAX7 expression on survival with other clinical characteristics, we performed multivariate Cox analysis. We determined the cutoff value of PAX7 expression based on the median, and statistical significance was defined as *p* < 0.05. To evaluate the diagnostic effect of PAX7 expression in distinguishing BRCA from healthy samples, ROC analysis was performed using the pROC package. AUC values ranged from 0.5 to 1.0, indicating a recognition rate of 50%–100%.

## 3 Results

### 3.1 PAX7 Expression in Pan‐Cancer

**FIGURE 1:**
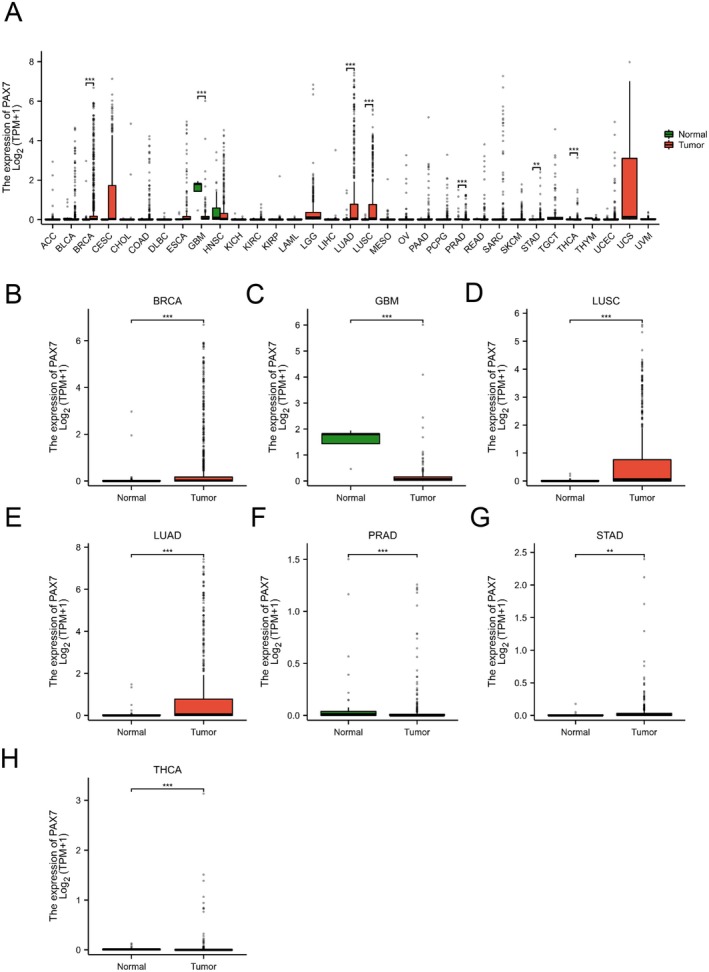
PAX7 expression in pan‐cancer. (A) Expression levels in PAX7 pan‐cancer samples versus paired normal samples. (B) PAX7 expression levels in normal samples and TCGA‐BRCA samples. (C) PAX7 expression levels in normal samples and TCGA‐GBM samples. (D) PAX7 expression levels in normal samples and TCGA‐LUSC samples. (E) PAX7 expression levels in normal samples and TCGA‐LUAD samples. (F) PAX7 expression levels in normal samples and TCGA‐PRAD samples. (G) PAX7 expression levels in normal samples and TCGA‐STAD samples. (H) PAX7 expression levels in normal samples and TCGA‐THCA samples. Between‐group analysis: Wilcoxon rank sum test; NS: *p* ≥ 0.05; *p < 0.05; **p < 0.01; ***p < 0.001.

After analysing the RNA‐seq data from the TCGA database consistently, we discovered that PAX7 exhibited varied expression levels in seven tumours when compared to normal samples (Figure 1A). In particular, PAX7 showed elevated levels in breast cancer (BRCA), lung adenocarcinoma (LUAD), lung squamous cell carcinoma (LUSC), gastric adenocarcinoma (STAD), and thyroid cancer (THCA), with a decrease in expression observed in prostate adenocarcinoma (PRAD) and glioma (GBM) (Figure 1B–H).

### 3.2 Prognostic Analysis of PAX7 in Pan‐Cancer

**FIGURE 2:**
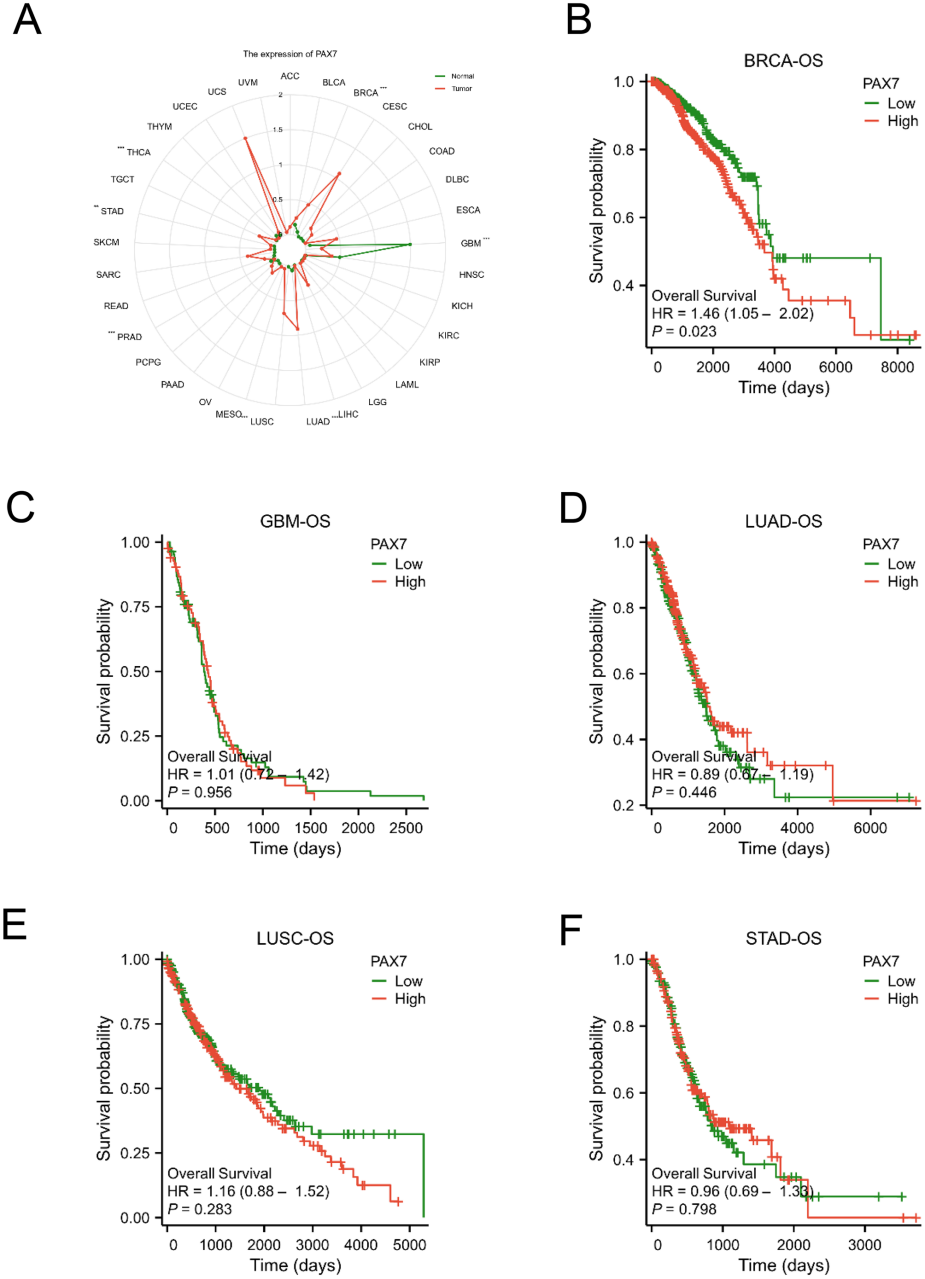
Prognostic analysis of PAX7 in pan‐cancer. (A) The radar chart indicated a correlation between PAX7 levels and seven types of cancer. (B‐F) Kaplan–Meier curves of OS in BRCA, GBM, LUAD, LUSC and STAD.

The radar chart indicated a correlation between PAX7 levels and seven types of cancer, such as breast cancer (BRCA), glioma (GBM), lung adenocarcinoma (LUAD), lung squamous cell carcinoma (LUSC), gastric adenocarcinoma (STAD), prostate adenocarcinoma (PRAD), and thyroid cancer (THCA) (Figure 2A). We utilised Kaplan–Meier analysis to investigate how PAX7 expression correlates with the prognosis of the mentioned cancers. PAX7 was specifically linked to the outcome of breast cancer, which is intriguing. Patients with high PAX7 levels in breast cancer had a significantly worse prognosis compared to those with low PAX7 levels, as indicated by the overall survival results (hazard ratio [HR] 1.46 (1.05–2.02); *p* = 0.023) (Figure 2B). In contrast, the overall survival results for glioma (GBM) showed a hazard ratio (HR) of 1.01 (0.72–1.42; *p* = 0.956), lung adenocarcinoma (LUAD) had a hazard ratio (HR) of 0.89 (0.67–1.19; *p* = 0.446), lung squamous cell carcinoma (LUSC) had a hazard ratio (HR) of 1.16 (0.88–1.52; *p* = 0.283), and gastric adenocarcinoma (STAD) had an overall survival result of (hazard ratio [HR] 0.96 (0.69–1.33); *p* = 0.798) (Figure 2B–F).

### 3.3 High PAX7 Expression Is Associated With Poor Prognosis in BRAC Patients

**FIGURE 3:**
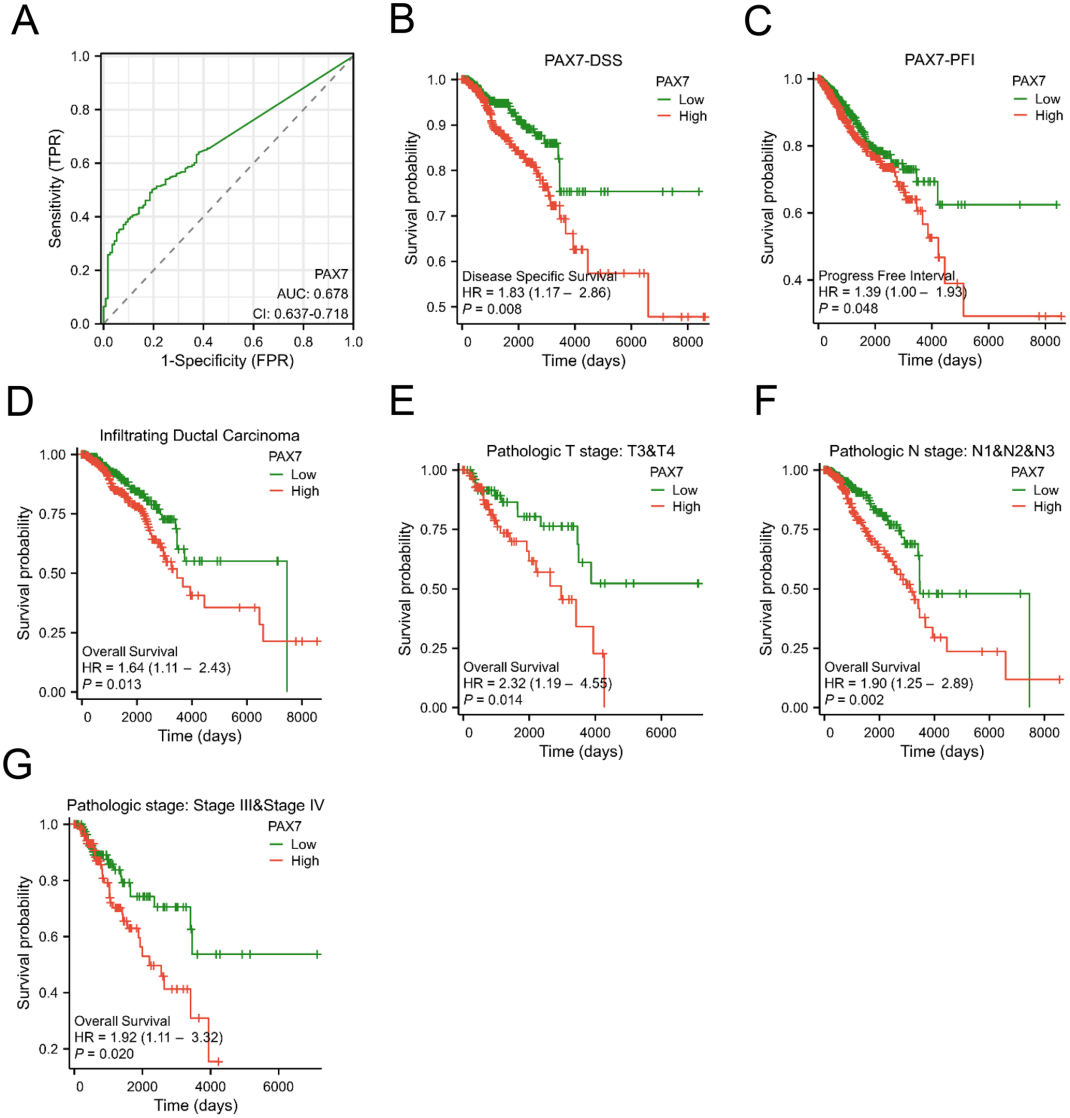
High PAX7 expression is associated with poor prognosis in BRAC patients. (A) ROC analysis of the diagnostic effect of PAX7 on BRCA. (B‐C) Kaplan–Meier curves of DSS and PFI in BRCA. (D‐G) Kaplan–Meier curves of BRCA patients in the subgroup.

Table 1 summarises the correlation between PAX7 expression and clinical features in BRAC patients in the TCGA database. ROC curve analysis was conducted to assess PAX7's capability in differentiating between BRAC patients and healthy individuals. The calculated AUC was 0.678 (CI: 0.637–0.718), indicating that PAX7 is a promising biomarker for breast cancer (Figure 3A). In our study, we conducted an analysis of the survival of breast cancer patients based on the expression of PAX7, revealing that elevated levels of PAX7 were linked to unfavourable outcomes in terms of disease‐specific survival (DSS) and progression‐free interval (PFI). The hazard ratio for DSS was 1.83 (1.17–2.86) with a *p*‐value of 0.008, while for PFI it was 1.39 (1.00–1.93) with a *p*‐value of 0.048. We also analysed the prognosis of PAX7 in BRAC subgroups. The Kaplan–Meier analysis indicated that PAX7 was linked to unfavourable outcomes in the infiltrating ductal carcinoma subgroup (HR = 1.64 (1.11–2.43), *p* = 0.013), T3 & T4 subgroup (HR = 2.32 (1.19–4.55), *p* = 0.014), N1 & N2 & N3 stage subgroup (HR = 1.90 (1.25–2.89), *p* = 0.002), and StageIII & StageIV subgroup (HR = 1.92 (1.11–3.32), *p* = 0.020) (Figure 3D–G). We conducted a univariate Cox proportional hazard regression analysis (Table 2) to assess the variables influencing overall survival (OS). The findings indicated that the expression of PAX7, along with the T stage, N stage, M stage, pathologic stage, and age, were determined to be indicators of unfavourable overall survival. After incorporating these variables into a multivariate Cox regression analysis, it was revealed that PAX7 emerged as a significant independent predictor of unfavourable overall survival (*p* = 0.049).

**TABLE 1:** Association between PAX7 expression and clinicopathologic features in BRCA samples from the TCGA.

Characteristics	Low expression of PAX7	High expression of PAX7	*p*
*n*	543	544	
Pathologic T stage, *n* (%)			0.206
T1 & T2	463 (42.7%)	446 (41.1%)	
T3 & T4	80 (7.4%)	95 (8.8%)	
Pathologic N stage, *n* (%)			0.499
N0	264 (24.7%)	252 (23.6%)	
N1 & N2 & N3	271 (25.4%)	281 (26.3%)	
Pathologic M stage, *n* (%)			0.073
M0	455 (49.2%)	450 (48.6%)	
M1	6 (0.6%)	14 (1.5%)	
Pathologic stage, *n* (%)			0.031
Stage I & Stage II	416 (39.1%)	385 (36.2%)	
Stage III & Stage IV	116 (10.9%)	146 (13.7%)	
Age, *n* (%)			0.881
≤ 60	300 (27.6%)	303 (27.9%)	
> 60	243 (22.4%)	241 (22.2%)	
PR status, *n* (%)			0.964
Negative	170 (16.4%)	172 (16.6%)	
Positive	345 (33.4%)	347 (33.6%)	
ER status, *n* (%)			0.053
Negative	133 (12.8%)	107 (10.3%)	
Positive	385 (37.1%)	412 (39.7%)	
HER2 status, *n* (%)			< 0.001
Negative	315 (43.9%)	245 (34.2%)	
Positive	47 (6.6%)	110 (15.3%)	
PAM50, *n* (%)			< 0.001
LumA	305 (29.1%)	259 (24.7%)	
LumB	73 (7%)	133 (12.7%)	
Her2	13 (1.2%)	69 (6.6%)	
Basal	133 (12.7%)	62 (5.9%)	

**TABLE 2:** Univariate and multivariate Cox's regression analysis of factors associated with OS in BRCA.

Characteristics	Total (*N*)	Univariate analysis		Multivariate analysis	
		Hazard ratio (95% CI)	*p*	Hazard ratio (95% CI)	*p*
PAX7	1086				
Low	542	Reference		Reference	
High	544	1.458 (1.053–2.017)	**0.023**	1.429 (1.001–2.039)	**0.049**
Pathologic T stage	1083				
T1 & T2	908	Reference		Reference	
T3 & T4	175	1.588 (1.096–2.301)	**0.014**	0.935 (0.558–1.566)	0.799
Pathologic N stage	1067				
N0	516	Reference		Reference	
N1 & N2 & N3	551	2.232 (1.563–3.189)	**< 0.001**	1.769 (1.138–2.752)	**0.011**
Pathologic M stage	925				
M0	905	Reference		Reference	
M1	20	4.266 (2.474–7.354)	**< 0.001**	2.127 (1.055–4.288)	**0.035**
Pathologic stage	1062				
Stage I & Stage II	800	Reference		Reference	
Stage III & Stage IV	262	2.367 (1.686–3.321)	**< 0.001**	1.776 (1.032–3.057)	**0.038**
Age	1086				
≤ 60	603	Reference		Reference	
> 60	483	2.024 (1.468–2.790)	**< 0.001**	2.131 (1.476–3.077)	**< 0.001**
PR status	1033				
Negative	342	Reference			
Positive	691	0.729 (0.521–1.019)	0.065		
ER status	1036				
Negative	240	Reference			
Positive	796	0.709 (0.493–1.019)	0.063		
HER2 status	717				
Negative	560	Reference			
Positive	157	1.593 (0.973–2.609)	0.064		

*Note:* The bold values in the table indicate that the differences are statistically significant.

### 3.4 Prognostic Predictive Model of PAX7 in BRAC

**FIGURE 4:**
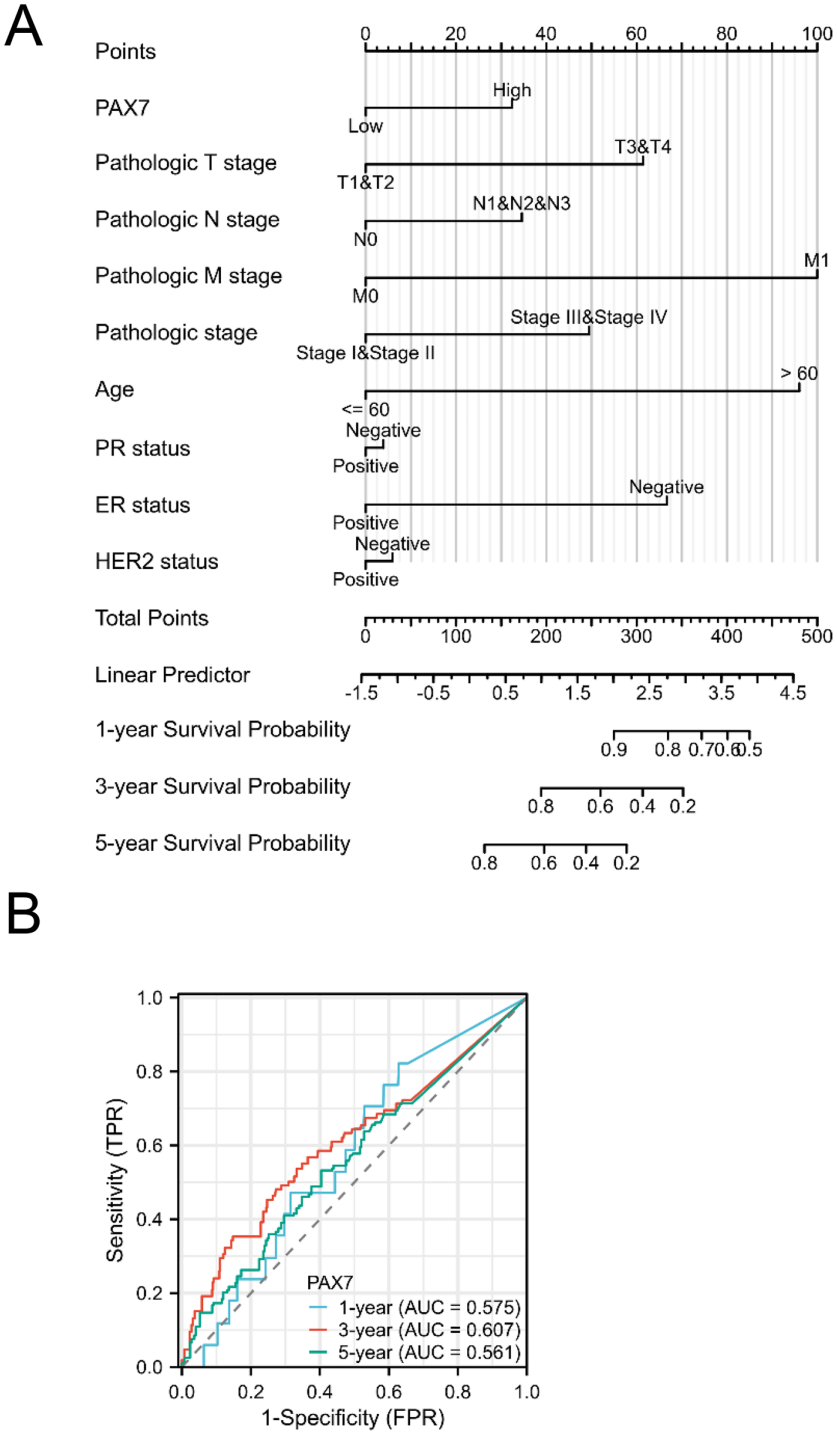
Prognostic predictive model of PAX7 in BRAC. (A)Nomogram for predicting 1‐, 3‐, and 5‐year OS probability in BRCA. (B) Prediction accuracy of nomogram calculated by AUC: 0.575 for 1‐year OS, 0.607 for 3‐year OS, and 0.561 for 5‐year OS.

To improve the forecast for BRAC patients, we created a nomogram with the RMS R package, including results from Cox regression analysis (Figure 4A). The nomogram showed strong predictive performance, achieving an AUC of 0.575 for 1‐year overall survival (OS), 0.607 for 3‐year OS, and 0.561 for 5‐year OS (Figure 4B).

### 3.5 Identification of DEGs in Breast Cancer Cell Line After PAX7 Knockdown

**FIGURE 5:**
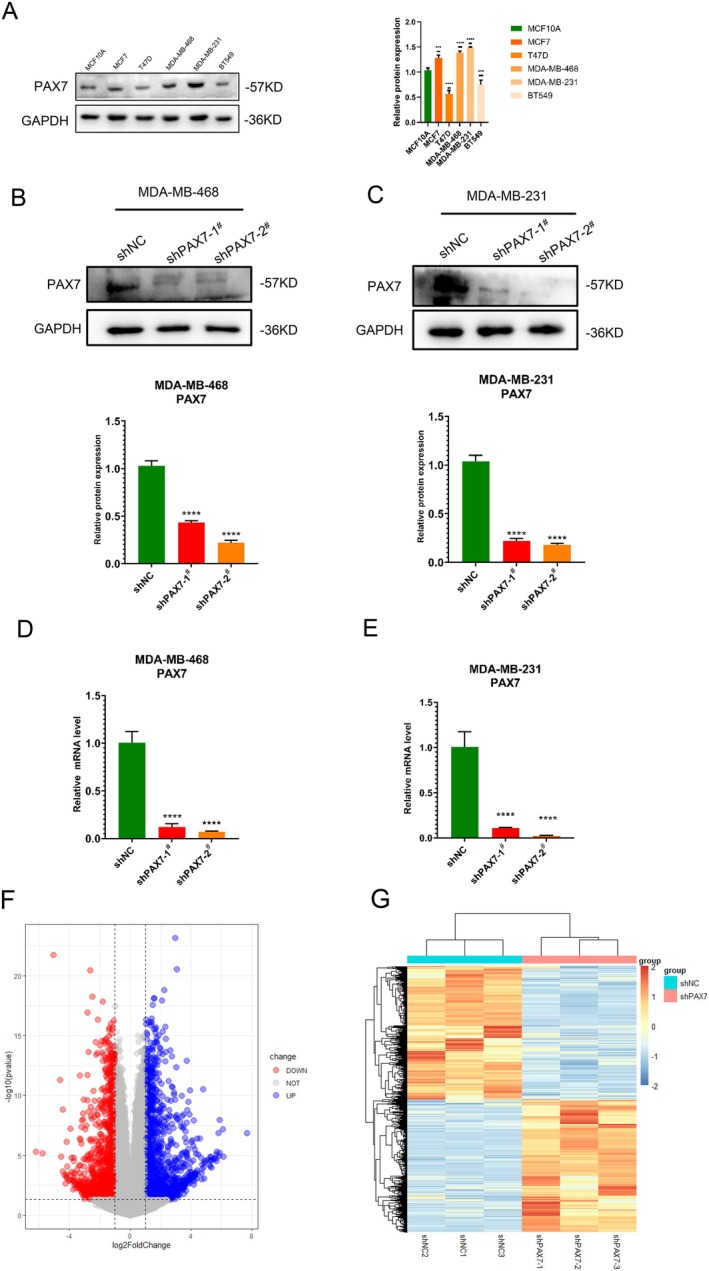
Identification of DEGs in breast cancer cell line after PAX7 knockdown. (A) Western blot analysis showed PAX7 expression in 5 breast cancer cell lines. (B) Western blot showed the expression of PAX7 in the PAX7 knockdown stable cell line MDA‐MB‐468. (C) Western blot showed the expression of PAX7 in the PAX7 knockdown stable cell line MDA‐MB‐231. (D) RT‐qPCR showed the expression of PAX7 in the PAX7 knockdown stable cell line MDA‐MB‐468. (E)Western blot showed the expression of PAX7 in the PAX7 knockdown stable cell line MDA‐MB‐231. (F) The volcano plot indicates that 1092 genes exhibited increased expression while 1070 genes showed decreased expression (LogFC ≥ 1, *p* < 0.05). (G) The heatmap displaying the top 50 genes with upregulated and downregulated expression levels. ***p < 0.001, ****p < 0.0001.

High levels of PAX7 in breast cancer were discovered to be associated with a negative prognosis after analysing clinical data from the TCGA database. Initially, we confirmed the presence of PAX7 in various typical breast cancer cell lines and observed that PAX7 expression is significantly elevated in MDA‐MB‐468 and MDA‐MB‐231 cell lines (Figure 5A). To confirm the role of PAX7 in breast cancer, we generated stable transfection cell lines with reduced PAX7 expression in MDA‐MB‐468 and MDA‐MB‐231 cells (Figure 5B–E) and conducted high‐throughput sequencing on the MDA‐MB‐231 cell line with PAX7 knockdown (Figure S1 display the quality control of high‐throughput sequencing data). The volcano plot indicates that 1092 genes exhibited increased expression while 1070 genes showed decreased expression (LogFC ≥ 1, *p* < 0.05) (Figure 5F), with the heatmap displaying the top 50 genes with upregulated and downregulated expression levels (Figure 5G).

### 3.6 Functional Enrichment Analysis of DEGs

**FIGURE 6:**
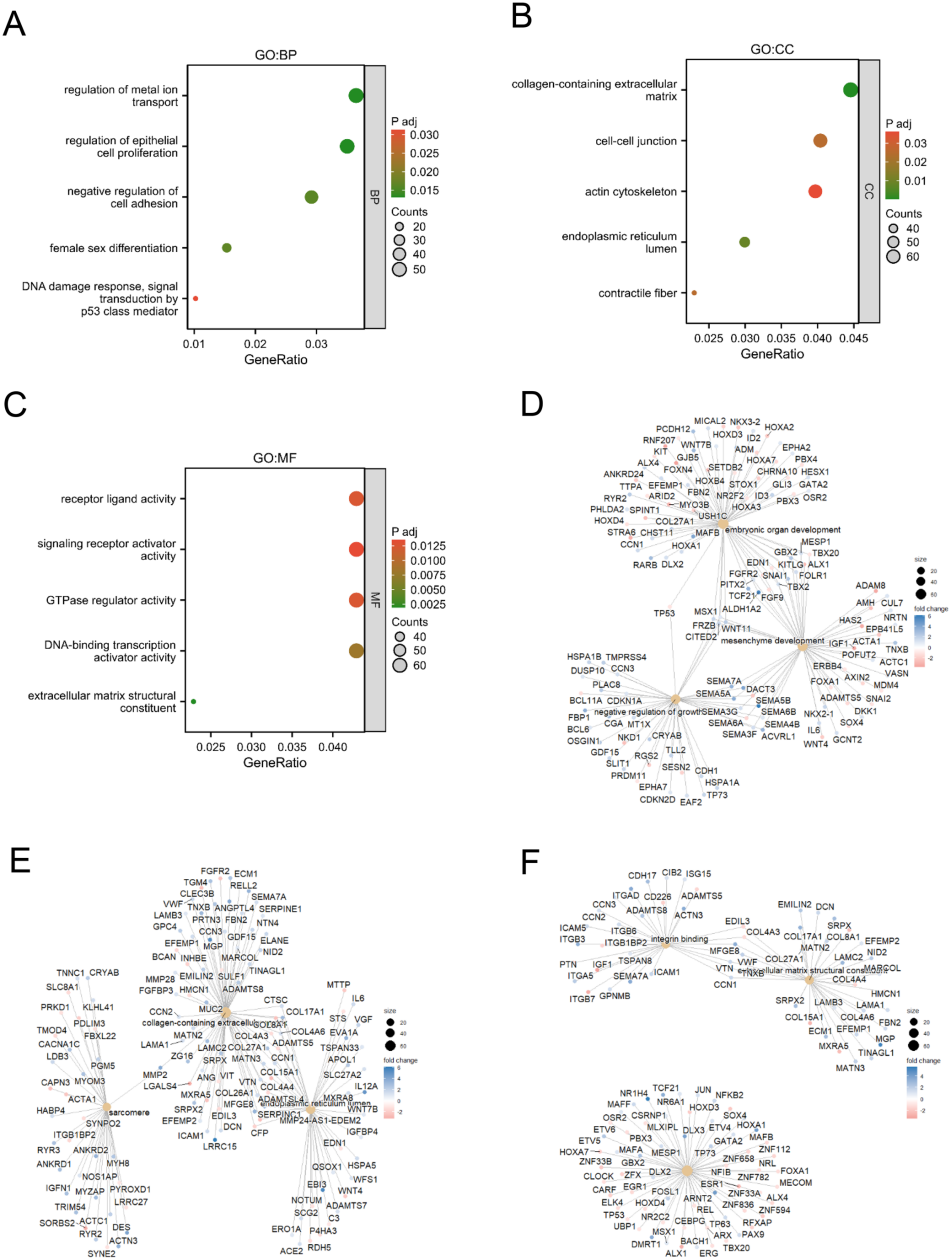
Functional enrichment analysis of DEGs. (A)GO enrichment analysis in the category of biological process (BP). (B) GO enrichment analysis in the category of cellular component (CC). (C) GO enrichment analysis in the category of molecular function (MF). (D) CNET map showed the key genes enriched in the BP. (E) CNET map showed the key genes enriched in the CC. (F) CNET map showed the key genes enriched in the MF.

**FIGURE 7:**
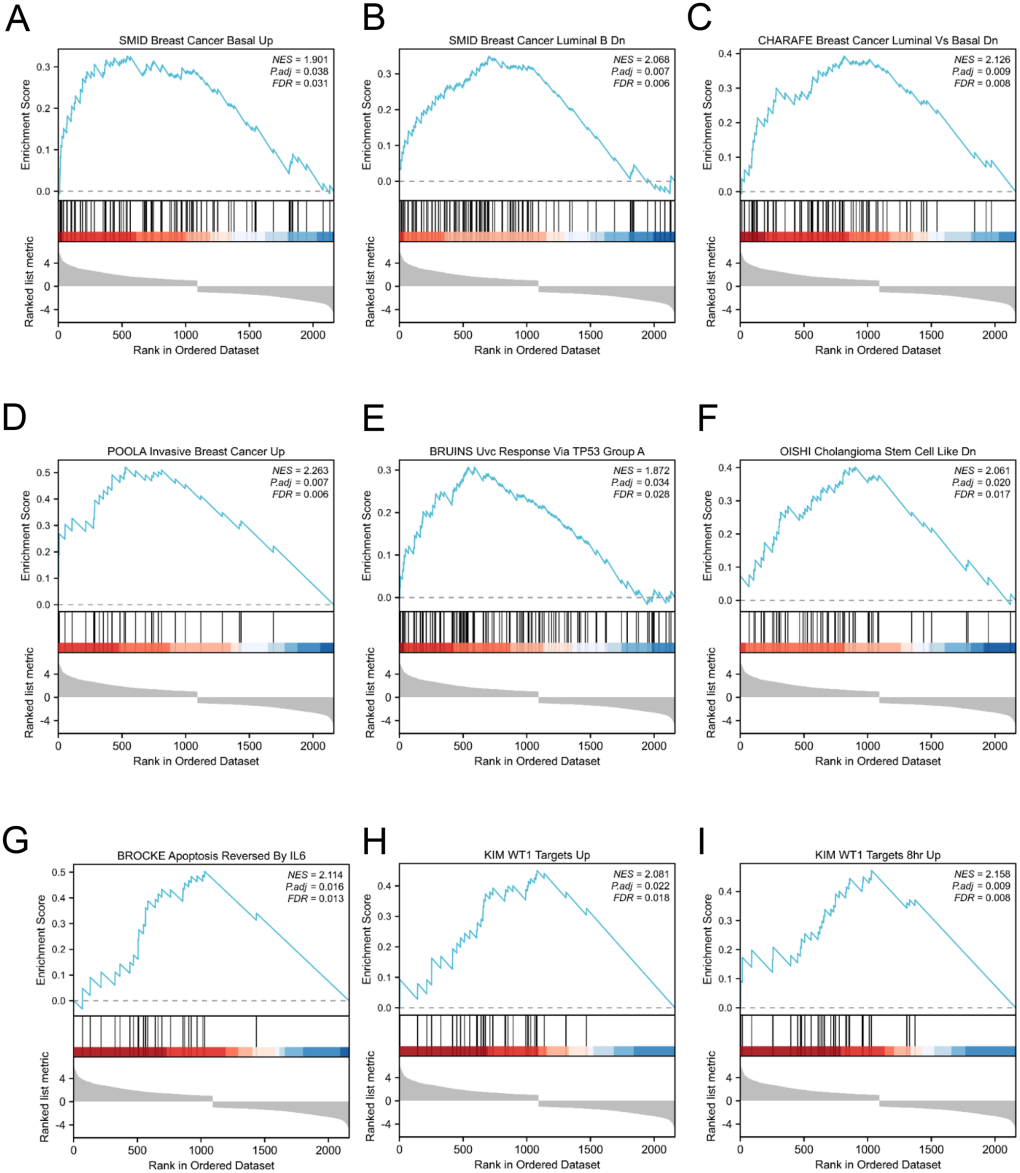
Enrichment plots from the gene set enrichment analysis (GSEA). (A–I) ES, enrichment score; NES, normalised ES; ADJ, adjusted *p*‐value.

The cluster Profiler package was utilised for conducting feature enrichment analysis on the aforementioned DEGs. Enriched biological processes (BP) consisted of controlling metal ion transportation, controlling epithelial cell growth, inhibiting cell adhesion, female gender differentiation, and responding to DNA damage (Figure 6A). The CNET map showed the key genes enriched in the BP (Figure 6D). The enriched cellular components (CC) included collagen‐containing extracellular matrix, cell–cell junction, actin cytoskeleton, endoplasmic reticulum lumen, and contractile fibre (Figure 6B). The CNET map showed the key genes enriched in the CC (Figure 6E). Furthermore, the enhanced molecular functions (MF) comprised activities such as receptor ligand, signalling receptor activator, GTPase regulator, DNA‐binding transcription activator, and extracellular matrix structure constituent (Figure 6C). The CNET map showed the key genes enriched in the MF (Figure 6F). Table S1 is a complete list of all enriched GO/KEGG terms. To better understand the biological pathways of PAX7 at different expression levels, we used GSEA to analyse DEGs. Through GSEA analysis, PAX7 was associated with breast cancer‐related signalling pathways and WNT signalling pathways (Figure 7A–I). Table S2 is a comprehensive list of all GSEA results.

### 3.7 PAX7 Knockdown Inhibited the Proliferation, Migration, and Invasion of Cancer Cells

**FIGURE 8:**
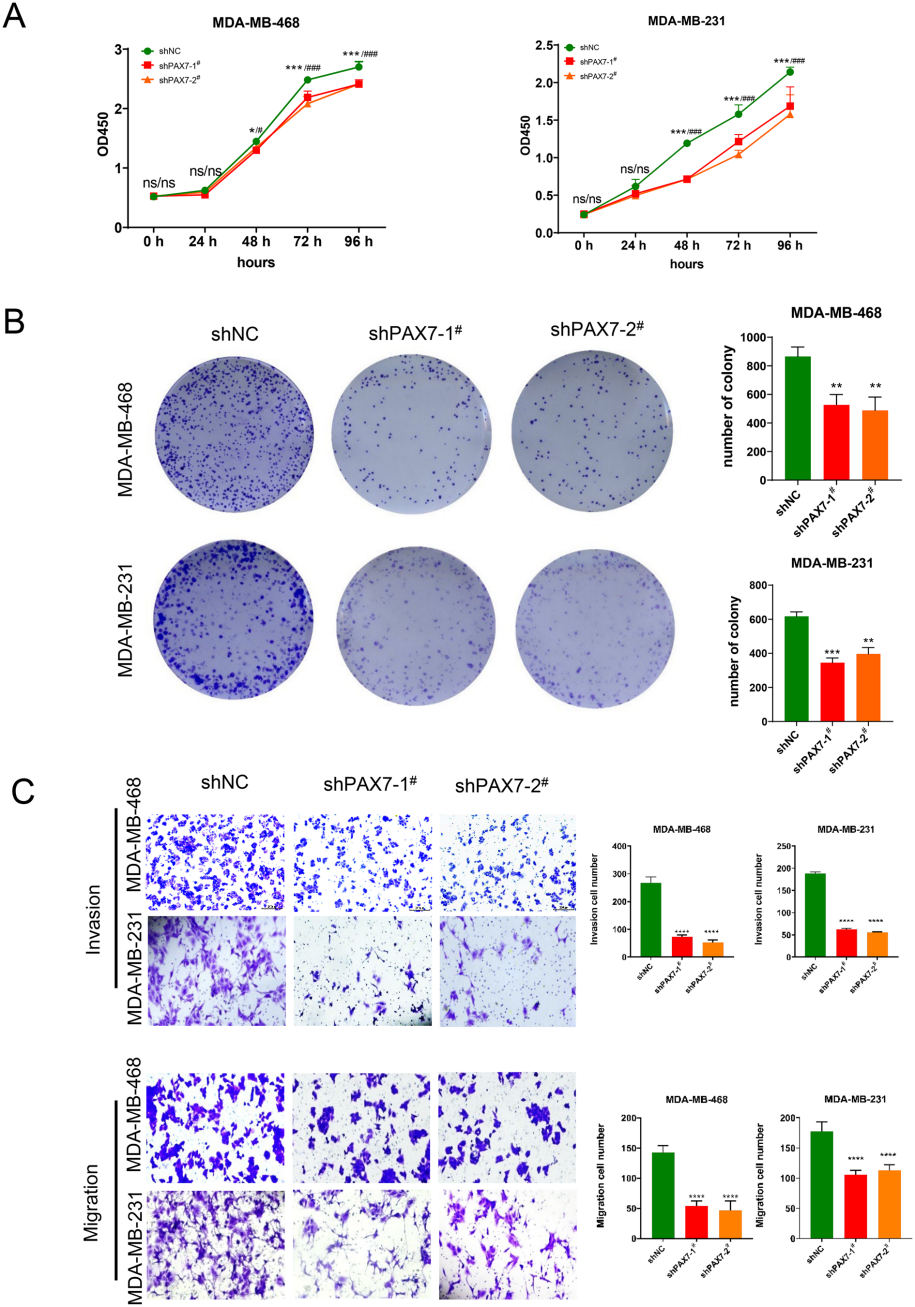
PAX7 knockdown inhibited the proliferation, migration, invasion of tumour cells. (A) CCK8 experiments indicated that suppressing PAX7 could impede the growth of cancer cells. (B) The clone formation experiment indicated that the knockdown of PAX7 could reduce the clone formation of cancer cells. (C) The transwell experiment indicated a reduction in the invasive and migratory capabilities of cancer cells following the suppression of PAX7. **p < 0.01, ***p < 0.001, ****p < 0.0001.

By analysing the results of high‐throughput sequencing, we found that the molecular function of PAX7 is mainly related to cell proliferation, invasion, and migration. Therefore, we performed CCK8 experiments on two PAX7 knockdown stable transfection cell lines. The findings indicated that suppressing PAX7 could impede the growth of cancer cells, as demonstrated in Figure 8A. The clone formation experiment showed that the knockdown of PAX7 could reduce the clone formation of cancer cells (Figure 8B). The Transwell study indicated a reduction in the invasive and migratory capabilities of cancer cells following the suppression of PAX7 (Figure 8C).

### 3.8 PAX7 Knockdown Inhibits Proliferation, Invasion, and Migration of Cancer Cells by Inhibiting the Wnt/β‐Catenin Signalling Pathway

**FIGURE 9:**
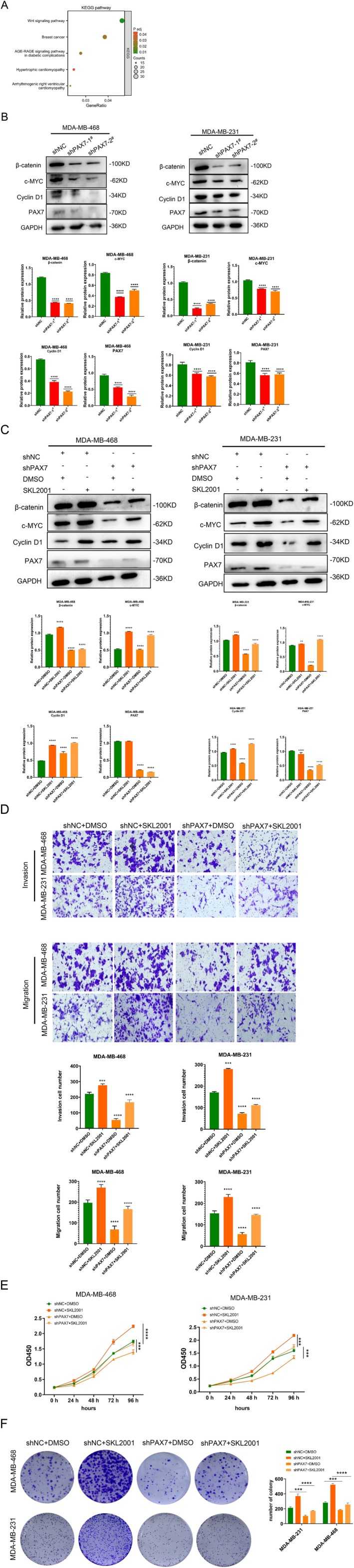
PAX7 knockdown inhibits breast cancer cell invasion and migration by inhibiting Wnt/β‐catenin signalling pathway. (A) KEGG pathway enrichment analysis. (B) Western Blot analysis revealed that reducing PAX7 levels suppressed the Wnt/β‐catenin signalling pathway in two cell types, resulting in reduced levels of β‐catenin, c‐MYC, and cyclin D1. (C) The Western Blot analysis indicated that SKL2001 was able to counteract the suppression caused by silencing PAX7 on the Wnt/β‐catenin signalling pathway and cyclingD1 protein levels. (D) Transwell experiments showed that SKL2001 could reverse the inhibitory effect of knocking down PAX7 on cancer cell migration and invasion. (E) CCK‐8 experiments showed that SKL2001 could reverse the inhibitory effect of PAX7 knockdown on cancer cell proliferation. (F) Colony formation assays showed that PAX7 knockdown significantly reduced the number of colonies, whereas SKL2001 treatment partially restored colony formation. **p < 0.01, ***p < 0.001, ****p < 0.0001.

Through KEGG analysis of the differentially expressed genes identified through high‐throughput sequencing, we discovered that these genes were predominantly enriched in pathways associated with breast cancer and Wnt signalling (Figure 9A). Western Blot analysis revealed that reducing PAX7 levels suppressed the Wnt/β‐catenin signalling pathway in two cell types, resulting in reduced levels of β‐catenin, c‐MYC, and cyclin D1 (Figure 9B). This suggests that suppressing PAX7 can block the activation of the Wnt/β‐catenin signalling pathway. To investigate the correlation between PAX7 and the Wnt/β‐catenin signalling pathway in breast cancer cells, we employed the Wnt/β‐catenin signalling pathway‐specific activator SKL2001 for additional research. The Western Blot analysis indicated that SKL2001 was able to counteract the suppression caused by silencing PAX7 on the Wnt/β‐catenin signalling pathway and cyclingD1 protein levels (Figure 9C). Transwell experiments showed that SKL2001 could reverse the inhibitory effect of knocking down PAX7 on cancer cell migration and invasion (Figure 9D). We performed CCK‐8 assay and colony formation assay to evaluate the effect of PAX7 on the proliferation of breast cancer cells. CCK‐8 results showed that PAX7 knockdown (shPAX7) significantly reduced cell proliferation compared with control (shNC) in both cell lines. Treatment with Wnt/β‐catenin pathway activator SKL2001 partially rescued the proliferation of PAX7 knockdown cells (shPAX7 + SKL2001), indicating that Wnt/β‐catenin activation counteracted the inhibitory effect of PAX7 knockdown on cell growth (Figure 9D). Similarly, colony formation assay results showed that PAX7 knockdown significantly reduced colony formation in MDA‐MB‐468 and MDA‐MB‐231 cells. SKL2001 treatment (shPAX7 + SKL2001) partially restored colony formation (Figure 9E).

The results of the study showed that inhibiting PAX7 could prevent the growth and invasion of breast cancer cells by blocking the Wnt/β‐catenin signalling pathway. PAX7 is expected to become a potential target for the treatment of breast cancer.

## 4 Discussion

Among women, breast cancer ranks as a prevalent form of cancer [1, 5]. Despite advancements in breast cancer treatment, challenges persist due to issues like side effects from targeted medications and the development of drug resistance [1, 2, 4]. Therefore, further understanding the molecular mechanism of breast cancer occurrence, accurately predicting its biological progression and prognosis, and exploring more effective drug targets are scientific issues that we urgently need to solve.

PAX7, a member of the PAX gene family, functions as a transcription factor [8, 9, 25]. Our findings presented in this study highlight the significant role of PAX7 in various cancers, with a particular emphasis on its expression and prognostic value in breast cancer. Examination of RNA‐seq information from the TCGA repository revealed variations in PAX7 expression among various cancer types, showing increased levels in BRCA, LUAD, LUSC, STAD, and THCA, while decreased levels were noted in PRAD and GBM.

Prognostic analysis revealed that while PAX7 expression is associated with seven types of cancer, its prognostic significance is most pronounced in breast cancer. High PAX7 expression in breast cancer correlates with worse overall survival (OS), suggesting its potential as a prognostic biomarker. The connection between PAX7 and negative outcomes is reinforced by examining disease‐specific survival (DSS) and progression‐free interval (PFI), especially within certain subcategories of breast cancer patients.

In addition, our research created a predictive model utilising a nomogram derived from Cox regression analysis, demonstrating strong predictive capability for the overall survival of breast cancer patients at 1, 3, and 5 years. The model, along with the DEGs found in high‐throughput sequencing data and their enrichment in important biological processes, gave a thorough insight into the function of PAX7 in breast cancer.

Elevated levels of PAX7 in breast cancer are associated with a negative outlook, supported by clinical information from the TCGA repository. MDA‐MB‐468 and MDA‐MB‐231 breast cancer cell lines show significant expression of PAX7. To explore the function of PAX7, stable transfection cell lines with PAX7 knockdown were created in these cell lines. Sequencing the knockdown PAX7 MDA‐MB‐231 cell line at a high rate showed notable alterations in gene expression, with 1092 genes showing increased expression and 1070 genes showing decreased expression (LogFC ≥ 1, *p* < 0.05). Following the analysis of enriched functions in genes with differential expression, several important biological processes, cellular components, and molecular functions were identified.

Experiments that reduced PAX7 expression in breast cancer cell lines (MDA‐MB‐468 and MDA‐MB‐231) revealed a significant influence on cell activity. Suppression of PAX7 hindered the growth, movement, and infiltration of cells, highlighting its importance in the behaviour of cancer cells. The findings from these molecular biology experiments align with the results of functional enrichment.

Prior research has demonstrated the significance of PAX7 as a crucial transcription factor in muscle formation and its involvement in different types of cancer, notably rhabdomyosarcoma (RMS) [8, 12, 13, 26]. Studies have shown that PAX7 is essential for the maintenance and proliferation of RMS cells [27, 28]. Dependence on PAX7 was evident in both cell line models of RMS and patient‐derived xenografts (PDX), where PAX7 knockdown resulted in significantly reduced tumour growth and viability [29]. These findings highlight the role of PAX7 in maintaining the ability of tumour cells to proliferate. Although no studies have yet proven the effect of PAX7 on the metastasis ability of tumour cells, we found that the functional enrichment of DEGs obtained by high‐throughput sequencing after PAX7 knockout showed that these DEGs mainly enriched biological processes and molecular functions related to tumour metastasis ability, such as negative regulation of cell adhesion, cell–cell junction, and actin cytoskeleton. While the primary focus has been on proliferation, there is also evidence suggesting that PAX7 may influence tumour cell invasion and migration. In FN‐RMS cells, reducing PAX7 expression results in tumours that are less invasive and have different histological features, indicating that PAX7 may be involved in maintaining the invasive properties of these cells [27, 30].

The mechanistic exploration revealed that PAX7 influences breast cancer through the Wnt/β‐catenin signalling pathway, resulting in reduced expression of β‐catenin, c‐MYC, and Cyclin D1 when PAX7 is suppressed. The ability to reverse these inhibitory effects with a Wnt/β‐catenin pathway activator (SKL2001) underscores the pathway's involvement.

The Wnt signalling pathway is crucial in the development of breast cancer, with particular emphasis on the significance of Wnt/β‐catenin pathway activation in tumour initiation and advancement [14, 15, 16, 31]. Breast cancer cells frequently exhibit alterations in the Wnt signalling pathway, such as gene mutations, amplifications, deletions, and methylation, along with post‐transcriptional modifications at both the mRNA and protein levels [15, 16, 18, 32, 33, 34]. Activation of intracellular β‐catenin occurs through the binding of Wnt to the Frizzled (Fzd) receptor and the low‐density lipoprotein receptor‐related protein (LRP) coreceptor [15, 33, 34]. In the absence of the Wnt ligand, β‐catenin is taken and broken down by the ‘destruction complex’; however, with the presence of the Wnt ligand, the destruction complex is blocked, leading to β‐catenin clustering and moving into the cell nucleus. This activates the transcription factors from the TCF/LEF family, starting the transcription of genes downstream [15, 16, 33, 34]. Breast cancer is associated with the abnormal activation of the Wnt/β‐catenin signalling pathway, which plays a crucial role in the proliferation, maintenance of stemness, and metastasis of cancer cells [15, 16, 19, 33]. The accumulation of β‐catenin in the nucleus is also associated with the development of breast cancer characteristics, influencing how it reacts to various therapies. The Wnt pathway activation plays a crucial role in triple‐negative breast cancer (TNBC) and basal‐like breast cancer (BLBC), according to studies [15, 19]. Mutations or silencing of E‐cadherin frequently coincide with these subtypes, causing β‐catenin to be released from the cell membrane and move into the cytoplasm, thereby enhancing the likelihood of it entering the nucleus and triggering downstream oncogenes [14, 32, 33]. The Wnt/β‐catenin pathway is crucial for the survival of breast cancer stem cells (BCSCs) and the spread of tumours [19, 31, 34, 35, 36]. Wnt signals enhance the self‐renewal and growth of breast cancer stem cells, as well as boost tumour invasiveness through the regulation of ‘stemness’ maintenance and metastatic potential in breast cancer cells [15, 16, 17, 19, 33]. Hence, controlling the Wnt signalling pathway could be a promising focus for treating breast cancer.

Although we have identified a link between PAX7 and the Wnt/β‐catenin pathway, its exact regulatory mechanism remains unclear. Studies have shown that PAX7 may interact with β‐catenin and chromatin remodelling factors to affect Wnt target genes such as Axin2 [37]. For example, PAX7 can inhibit β‐catenin activity by recruiting histone deacetylases (HDAC1), thereby limiting transcriptional activation of Wnt targets [38]. In turn, Wnt signalling can regulate PAX7, as Wnt3a has been shown to downregulate PAX7 protein levels and promote muscle progenitor differentiation [39]. This reciprocal feedback may also apply to breast cancer, where PAX7 can regulate components of the Wnt/β‐catenin pathway, such as β‐catenin, Wnt ligands, or inhibitors such as GSK3β, while modulating chromatin accessibility or recruiting coregulators. Further studies are needed to elucidate the role of PAX7 in breast cancer and its interaction with Wnt/β‐catenin signalling, which may underlie key processes such as proliferation, migration, and invasion.

**FIGURE 10:**
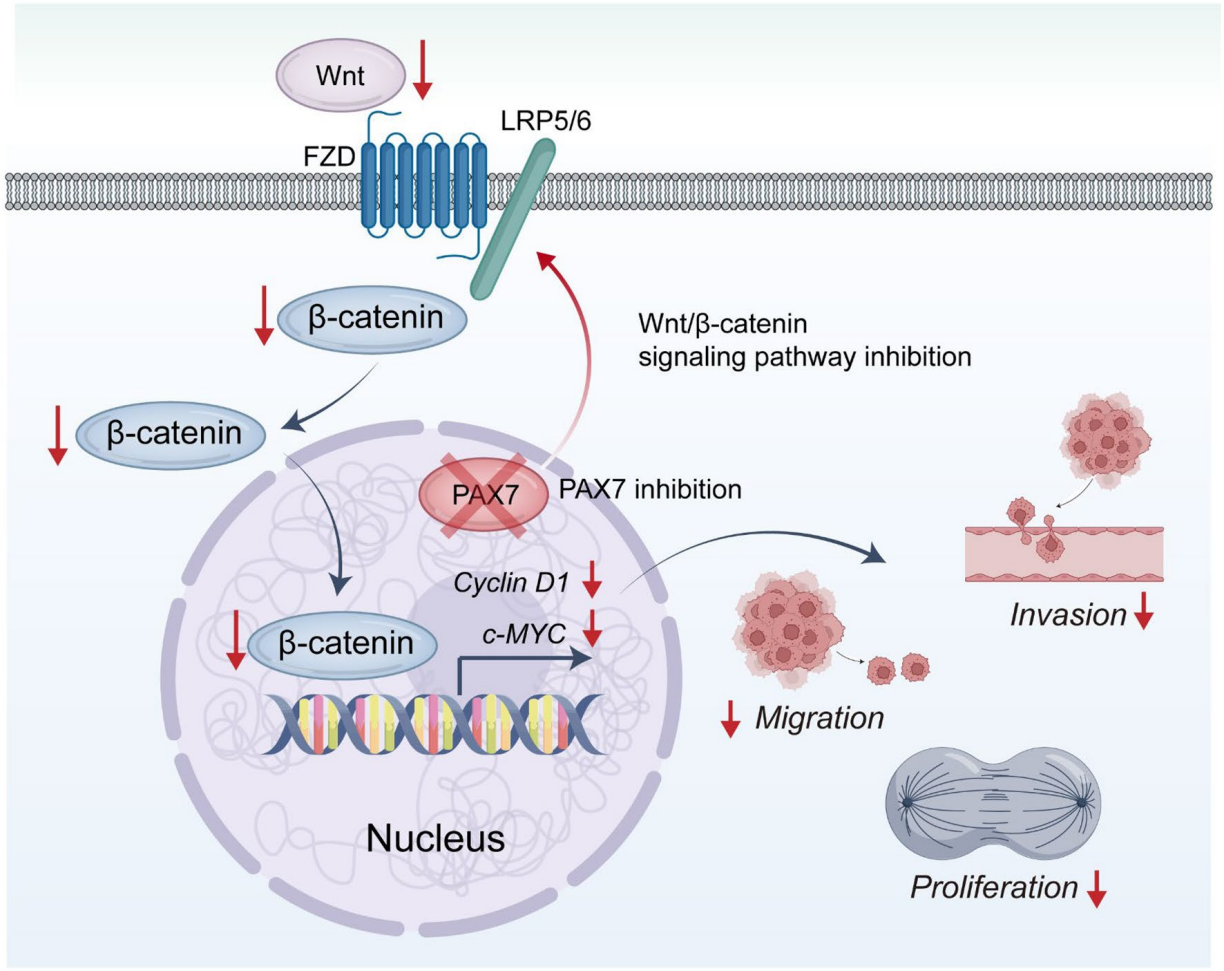
PAX7 knockdown inhibits the Wnt/β‐catenin signalling pathway and inhibits breast cancer proliferation and metastasis.

Similarly, while bioinformatics analysis provides a solid foundation for identifying PAX7 as a potential prognostic biomarker and therapeutic target, reliance on publicly available datasets introduces potential biases and incomplete clinical annotations. Future studies should use larger, more diverse, and independent cohorts to validate these findings. Similarly, while in vitro experiments showed that PAX7 knockdown inhibited breast cancer cell proliferation, migration, and invasion, these experiments were performed in limited cell lines, and the heterogeneity of breast cancer requires validation across different molecular subtypes. Further exploration of the role of PAX7 in breast cancer progression, particularly its regulation of the Wnt/β‐catenin pathway, is critical. We have initiated related studies, including single‐cell sequencing to analyse its impact on the immune microenvironment of breast cancer, and the results will be published soon. In summary, because PAX7 greatly influences the outlook and is involved in important cellular functions controlled by the Wnt/β‐catenin signalling pathway (Figure 10), it could serve as a crucial biomarker and potential target for the treatment of breast cancer. Further studies are warranted to explore PAX7‐targeted therapies and their potential clinical applications in breast cancer treatment.

## Author Contributions

**Qidong Ge:** data curation (equal), funding acquisition (equal), writing – original draft (equal). **Wei Zhang:** methodology (equal), visualization (equal). **Chao Li:** data curation (equal), methodology (equal). **Xinlin Li:** data curation (equal), methodology (equal). **Zhen Wang:** conceptualization (equal), data curation (equal), writing – review and editing (equal). **Xujun Li:** conceptualization (equal), writing – review and editing (equal).

## Ethics Statement

The data involved in this article is based on public databases and cell experiments, and does not involve animals or humans. The experimental plan of this article has been approved by the Committee of Ningbo Second Hospital.

## Conflicts of Interest

The authors declare no conflicts of interest.

## Supporting information


**Figure S1.** The quality control of high‐throughput sequencing data. (A) The PCA diagram shows the distribution of shNC and shPAX7 samples. (B) Histogram showing quality assessment of shNC and shPAX7 samples.

**Table S1.** All GO_KEGG results.

**Table S2.** All GSEA results.

## Data Availability

The datasets generated/analysed during the current study are available.
