# Corrigendum to “The Role of **PAX7** in Breast Cancer Prognosis and Its Mechanistic Involvement in the Wnt/β‐Catenin Pathway”

**DOI:** 10.1111/jcmm.71176

**Published:** 2026-05-12

Ge, Q., W. Zhang, C. Li, X. Li, Z. Wang, and X. Li. 2025. “The Role of PAX7 in Breast Cancer Prognosis and Its Mechanistic Involvement in the Wnt/β‐Catenin Pathway.” *Journal of Cellular and Molecular Medicine* 29: e70602. https://doi.org/10.1111/jcmm.70602.

In the originally published version of this article, two representative images in Figure 9D were inadvertently misused during figure assembly. Specifically, in the invasion assay for MDA‐MB‐468 cells, the images corresponding to the shNC+DMSO group and the shPAX7+DMSO group were incorrect. This error occurred because of confusion in file organization during the preparation of the final figures.

After careful re‐examination of the original raw data and experimental records, we identified the correct representative images for these two groups.

The corrected Figure 9D has been submitted as a separate figure file attachment. This correction is limited to the representative image presentation and does not affect the underlying experimental data, quantitative analysis, results, interpretation, or conclusions of the article. All other parts of Figure 9D remain unchanged.

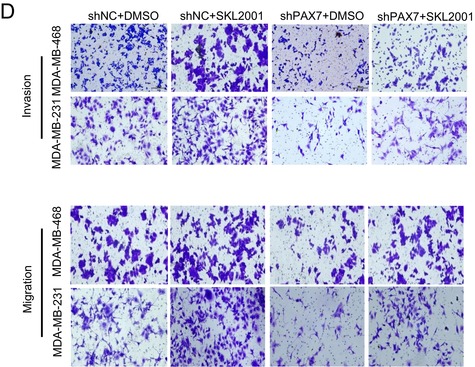

We apologise for this error.

