# Supplementary figures and images for: The Role of PAX7 in Breast Cancer Prognosis and Its Mechanistic Involvement in the Wnt/β‐Catenin Pathway
Source: J Cell Mol Med. 2025 May 15;29(10):e70602. doi: 10.1111/jcmm.70602 (PMC12079090; doi:10.1111/jcmm.70602)

Figure S1. The quality control of high-throughput sequencing data.

A

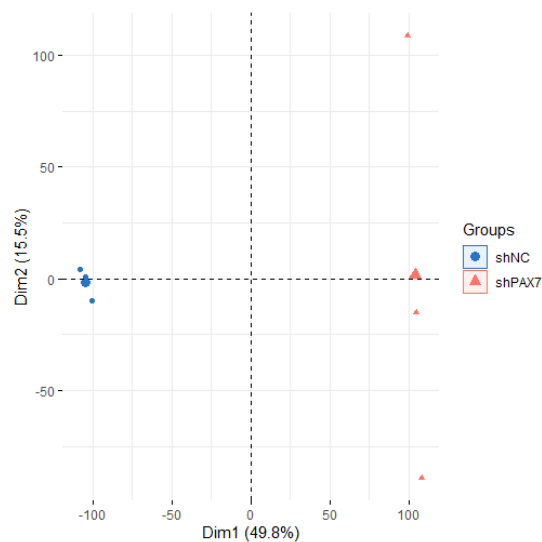

B

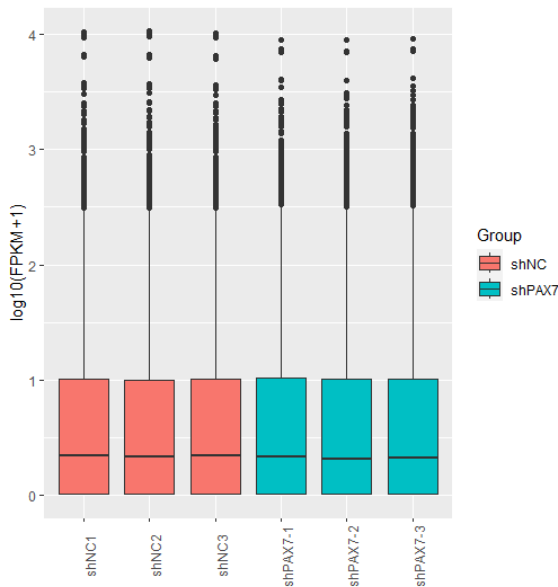

Supplement: Supplementary file 1 — Figure S1. The quality control of high‐throughput sequencing data. (A) The PCA diagram shows the distribution of shNC and shPAX7 samples. (B) Histogram showing quality assessment of shNC and shPAX7 samples. [file JCMM-29-e70602-s002.pdf]
